# Case Report: Inherited chromosomally integrated HHV-6B in a pediatric medulloblastoma patient with encephalitis

**DOI:** 10.3389/fimmu.2025.1615359

**Published:** 2025-07-31

**Authors:** Scott Sun, Konstance Knox, Alexander Romashko, Madhu Kundu, Haiying Zhu, Meei-Li Huang, Denise M. Malicki, John R. Crawford

**Affiliations:** ^1^ Rady Children’s Hospital, San Diego, CA, United States; ^2^ Renaissance School of Medicine, Stony Brook University, Stony Brook, NY, United States; ^3^ Coppe Healthcare Solutions, Inc., Waukesha, WI, United States; ^4^ Department of Laboratory Medicine and Pathology, Virology Division, University of Washington, Seattle, WA, United States; ^5^ Department of Pathology, University of California San Diego, La Jolla, CA, United States; ^6^ Department of Pediatrics and Neurology, University of California Irvine, Irvine, CA, United States; ^7^ Children’s Hospital Orange County, Orange, CA, United States

**Keywords:** HHV-6, HHV-6B, IciHHV-6, ciHHV-6, eHHV-6, chromosomally integrated HHV-6, medulloblastoma, encephalitis

## Abstract

**Background:**

Human herpesvirus 6B (HHV-6B) is associated with various central nervous system (CNS) disorders, particularly in immunocompromised patients. We present a rare case of inherited chromosomally integrated HHV-6B (iciHHV-6B), also referred to as endogenous HHV-6B (eHHV-6B) discovered during the workup for encephalitis in a child with relapsed non-WNT/non-SHH medulloblastoma.

**Case presentation:**

A preschool aged female was treated in infancy for non-WNT, non-SHH medulloblastoma with high dose chemotherapy and autologous stem cell transplant. She had leptomeningeal recurrence 6 months after transplant and received salvage therapy with high dose craniospinal proton therapy. Six months following radiation, she developed high fever, acute encephalopathy and seizures. Neuroimaging revealed left posterior temporal gyral edema, while extensive infectious/paraneoplastic/autoimmune workup demonstrated markedly elevated HHV-6B viral loads, consistent with inherited chromosomally integrated HHV-6B (iciHHV-6B) which was confirmed through blood digital droplet PCR analysis and PCR-based nail clipping analysis demonstrating paternal inheritance. Retrospective immunohistochemical analysis of the original medulloblastoma revealed HHV-6 late structural glycoprotein H antigens in peritumoral lymphocytes. Serum HHV-6 U100 glycoprotein mRNA detection was consistent with iciHHV-6B. Treatment included intravenous immunoglobulin (IVIG) and ganciclovir followed by a 12-month course of IVIG monotherapy with clinical improvement.

**Conclusion:**

Our case highlights the diagnostic challenges, controversies and clinical implications of ici-HHV-6/eHHV-6 in immunocompromised patients with encephalitis, where qualitative CSF viral detection alone may be insufficient for accurate diagnosis and cannot identify ici-HHV-6 status which complicates treatment decisions. This case demonstrates the importance of multimodal testing including chromosomal integration analysis in patients with high HHV-6 viral loads.

## 1 Introduction

Human herpesvirus 6 (HHV-6) is a member of the ß-Herpesviridae subfamily, comprising two variants, HHV-6A and HHV-6B, both of which have seroprevalence approaching 100% in most developed countries (1–4). Despite sharing over 90% sequence homology, these variants exhibit distinct tissue distribution and pathogenicity (5, 6). Primary infection typically occurs before age 3, with HHV-6B being the predominant cause. Childhood HHV-6 infection commonly presents as febrile illness, occasionally manifesting as roseola infantum (exanthem subitum), for which it is the etiological cause.

Following primary infection, HHV-6 establishes latency in various tissues, including the central nervous system (CNS), and can reactivate under conditions of immunosuppression (4, 7–11). HHV-6, particularly the B variant, has been implicated in various CNS diseases including encephalitis, multiple sclerosis, and febrile seizures (1, 12–14). Additionally, HHV-6 has been detected in CNS pediatric and adult brain tumors including glioma and medulloblastoma (15–17), especially in patients with chromosomally integrated HHV-6 (ci-HHV-6).

Chromosomal integration of HHV-6 is unique among the human herpesvirus family and occurs in approximately 1% of the population (8, 18, 19). The ci-HHV-6, also referred to as eHHV-6, viral genome integrates into telomeric regions of the host chromosomes and is subsequently transmitted in a Mendelian manner. This integration can result in persistently high viral levels in blood and other tissues, complicating diagnosis and potentially influencing clinical outcomes. The reactivation of HHV-6 in immunocompromised patients, particularly those undergoing cancer treatment, presents significant challenges in diagnosis and management (4, 10, 11).

Medulloblastoma is the most common malignant brain tumor in children, accounting for approximately 10% of all childhood brain and CNS tumors (20). Recent advancements in treatment have improved outcomes with 5-year survival rates exceeding 70% depending on the subtype. However, patients remain at risk for various complications, including infections and treatment-related neurotoxicity. Seizures are a presenting symptom in nearly half of all adult brain tumor cases and develop later in disease course as well (21). In metastatic brain tumors, seizures complicate up to 35% of patients. While a direct causal link between HHV-6 infection (latent, active, or chromosomally integrated) and brain tumors has not been established, a recent meta-analysis showed a high association of HHV-6 with primary brain tumors, suggesting a potential role in their development (9, 15, 22). The relationship between HHV-6 and CNS malignancies is an area of ongoing research, particularly with ci-HHV-6 (3, 9, 15, 22).

Here we present a pediatric patient with a history of medulloblastoma who developed encephalitis post-high dose radiation therapy. Subsequent studies revealed inherited chromosomally integrated HHV-6B (ici-HHV-6B) with suggestive evidence of viral RNA detection from blood and viral antigen detection in lymphocytes from the original tumor, though the temporal and causal relationships remain unclear. This case highlights the clinical significance of ici-HHV-6B/eHHV-6B in the context of encephalitis and CNS malignancies worthy of further study.

## 2 Case report

**Figure 1:**
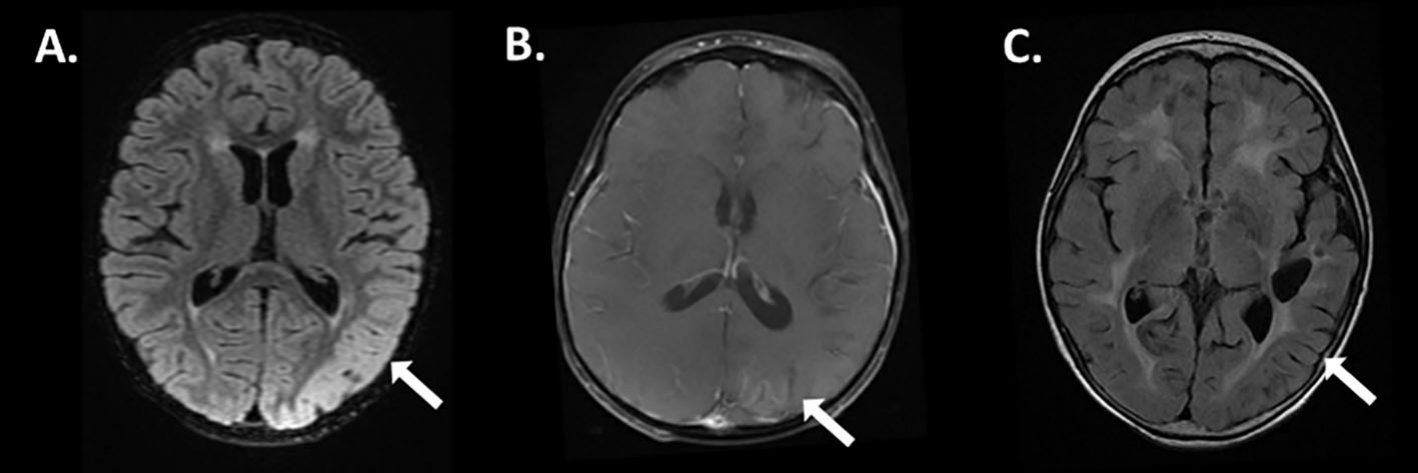
Magnetic resonance imaging features of ici-HHV-6B associated with encephalitis. 3D cube fluid attenuation inversion recovery (FLAIR) sequences at diagnosis revealed hyperintensity with gyral edema in the left posterior temporal lobe (**A**, arrow), associated with minimal enhancement on post-T1 gadolinium sequences (**B**, arrow). Six months after resolution of encephalitis resultant volume loss is appreciated on FLAIR sequences (**C**, arrow).

A female child with a history of recurrent non-WNT/non-SHH medulloblastoma presented 6 months following treatment with high dose craniospinal proton radiation with fever, acute encephalopathy and seizures. Her initial medulloblastoma chemotherapy regimen as an infant included cisplatin, cyclophosphamide, etoposide, vincristine, and high dose methotrexate followed by conditioning with carboplatin and thiotepa and triple tandem autologous stem cell transplant. Ten months following completion of stem cell therapy, she developed leptomeningeal brain and spinal metastatic disease and underwent high dose craniospinal proton salvage therapy. During proton therapy, she experienced complications including recurrent vomiting and somnolence, requiring a one-month course of high-dose steroid therapy. One week prior to admission, she developed upper respiratory symptoms associated with low grade fever and was treated with a course of oral antibiotics. Her symptoms evolved to include headache, recurrent vomiting, and progressive decline in mental status. On arrival she was febrile (104F) and neurologic examination revealed severe encephalopathy, with withdrawal to painful stimulation only and increased tone in the right upper extremity associated with hyperreflexia. Computed tomography demonstrated no acute findings (not shown) and routine complete blood count, serum chemistry panel was normal. Lumbar puncture revealed a glucose of 24mg/dL, protein 65mg/dL, white blood count 2/mm3, red blood count 0/µL and negative cytology. She started empiric antibiotics and acyclovir which were discontinued following negative cultures and HSV1/2 polymerase chain reaction (PCR) testing. Continuous electroencephalogram demonstrated diffuse delta slowing with electroclinical seizures arising from the left posterior region (not shown) which resolved following intravenous levetiracetam. MRI brain demonstrated diffuse posterior temporal/occipital gyral edema without evidence of tumor recurrence (**Figure 1A**) and negative MRI spine (not shown). The differential diagnosis included disease recurrence vs viral/autoimmune encephalitis, and an extensive panel of testing was performed as shown in **Table 1** to exclude infectious and autoimmune/paraneoplastic etiologies which only revealed positive HHV-6 PCR from the CSF. While MOG antibody-associated disease can be considered given the cortical encephalitis features and IVIG responsiveness, testing was not yet commercially available at the time of diagnosis, representing an important diagnostic consideration for future cases. Furthermore, HHV-6 IgM/IgG titers in CSF were not performed, which could have better helped distinguish active infection from incidental ici-HHV-6 findings. The patient showed recovery over the course of 7 days and was discharged home on oral levetiracetam without further clinical or electrographic seizures.

**Table 1:** Initial and confirmatory studies.

Initial investigations				
Studies	Testing	Source	Reference ranges/units	Results
Paraneoplastic Autoantibody Evaluation	Anti-neuronal Nuclear Antibody-Type 1 (ANNA-1)	Blood	Negative at <1:2	Negative
	Anti-neuronal Nuclear Antibody-Type 2 (ANNA-2)			Negative
	Anti-neuronal Nuclear Antibody-Type 3 (ANNA-3)			Negative
	Anti-Glial Nuclear Ab, Type 1			Negative
	Purkinje Cell Cytoplasmic Antibody, Type 1			Negative
	Purkinje Cell Cytoplasmic Antibody, Type 2			Negative
	Purkinje Cell Cytoplasmic Antibody, Type Tr			Negative
	Amphiphysin Antibody CSF			Negative
	CRMP-5 IGG CSF			Negative
Meningitis/Encephalitis PCR Panel	Herpes Simplex Virus Type 1	CSF	N/A	Negative
	Herpes Simplex Virus Type 2			Negative
	Varicella Zoster Virus			Negative
	Epstein-Barr Virus			Negative
	Cytomegalovirus			Negative
	Human Herpesvirus Type 6			**Positive**
	Enterovirus sp.			Negative
Respiratory Viral PCR Panel	Influenza A	NP/Throat Swab	N/A	Negative
	Influenza B			Negative
	Adenovirus			Negative
	Human Metapneumovirus			Negative
	Respiratory Syncytial Virus			Negative
	Enterovirus sp			Negative
	Mycoplasma			Negative
	Parainfluenza 1			Negative
	Parainfluenza 2			Negative
	Parainfluenza 3			Negative
IgM Antibody - Enzyme Immunoassay	Saint Louis Encephalitis Virus	Serum	N/A	Negative
	Western Equine Encephalitis Virus			Negative
	West Nile Virus			Negative
	Herpes Simplex Virus			Negative
	Varicella Zoster Virus			Negative
	Human Herpesvirus Type 6 (HHV-6)			Negative
	Epstein Barr Virus (immunofluorescence assay)			Negative
	Measles Virus			Negative
	Mycoplasma sp.			Negative
	Chlamydia sp.			Negative
	Influenza Type A			Negative
	Influenza Type B			Negative
	Adenovirus			Negative
Other Infectious/Autoimmune Studies	NMDA Receptor Antibody Test	CSF	N/A	Negative
	QuantiFERON-TB Gold test	Blood	N/A	Negative
	Coccicoides Antibody	CSF	N/A	Negative
	Bartonella henselae & Bartonella quintana Antibodies, IgM & IgG	CSF	N/A	Negative
	Cryptococcus Antigen Screen	CSF	N/A	Negative

Ici HHV6-B Confirmatory Testing				
Studies	Testing	Source	Reference Ranges/Units	Results
Initial HHV6 Exploration	HHV6 Quantitative Sensory Testing	Blood	<500 copies/mL	>2000000 copies/mL
	HHV6B DNA Quantitative PCR	Plasma	3.0-6.0 log copies/mL	3.7 log copies/mL
	HHV6 DNA Quantitative PCR	CSF	<500 copies/mL	1842 copies/mL
Chromosomal HHV-6B Workup	HHV-6 DNA PCR, Father	Nail Clippings	N/A	HHV-6B detected
	HHV-6 DNA PCR, Mother			HHV-6B not detected
	HHV6 DNA PCR, Patient			HHV-6B detected
	HHV6 DNA, RT PCR	Plasma	<500 copies/mL	1010502 copies/mL
	HHV6B U100 (glycoprotein Q) mRNA RT-qPCR	Blood	N/A	35.6 copies/10mL
	HHV6B Droplet Digital Quantitative PCR	Blood	N/A	1.12E+05 copies/cell

Bold indicates a positive result in Initial investigations section.

Four weeks post discharge she presented again with fever (101.3F) and encephalopathy necessitating admission. Testing revealed positive serum/CSF HHV-6 PCR, however quantitative PCR from the blood, plasma, and CSF demonstrated a marked elevation in HHV-6B viral load and ci-HHV-6B was suspected based on the copy number elevation (**Table 1**). Repeat MRI demonstrated only mild left parietal/occipital post-gyral enhancement (**Figure 1B**) and resolution of gyral edema. EEG revealed diffuse slowing without seizures (not shown). She was treated with 2g/kg of intravenous immunoglobulin for concerns of autoimmune encephalitis and a 14-day course of ganciclovir for concerns of HHV-6B reactivation, thought distinguishing between ici-HHV-6B or exogenous infection remained challenging given the lack of pre-illness baseline viral load data, with clinical improvement. The patient was subsequently treated with a 12-month course of monthly IVIG without subsequent hospitalization. Repeat MRI demonstrated left posterior parietal/occipital atrophy (**Figure 1C**).

**Figure 2:**
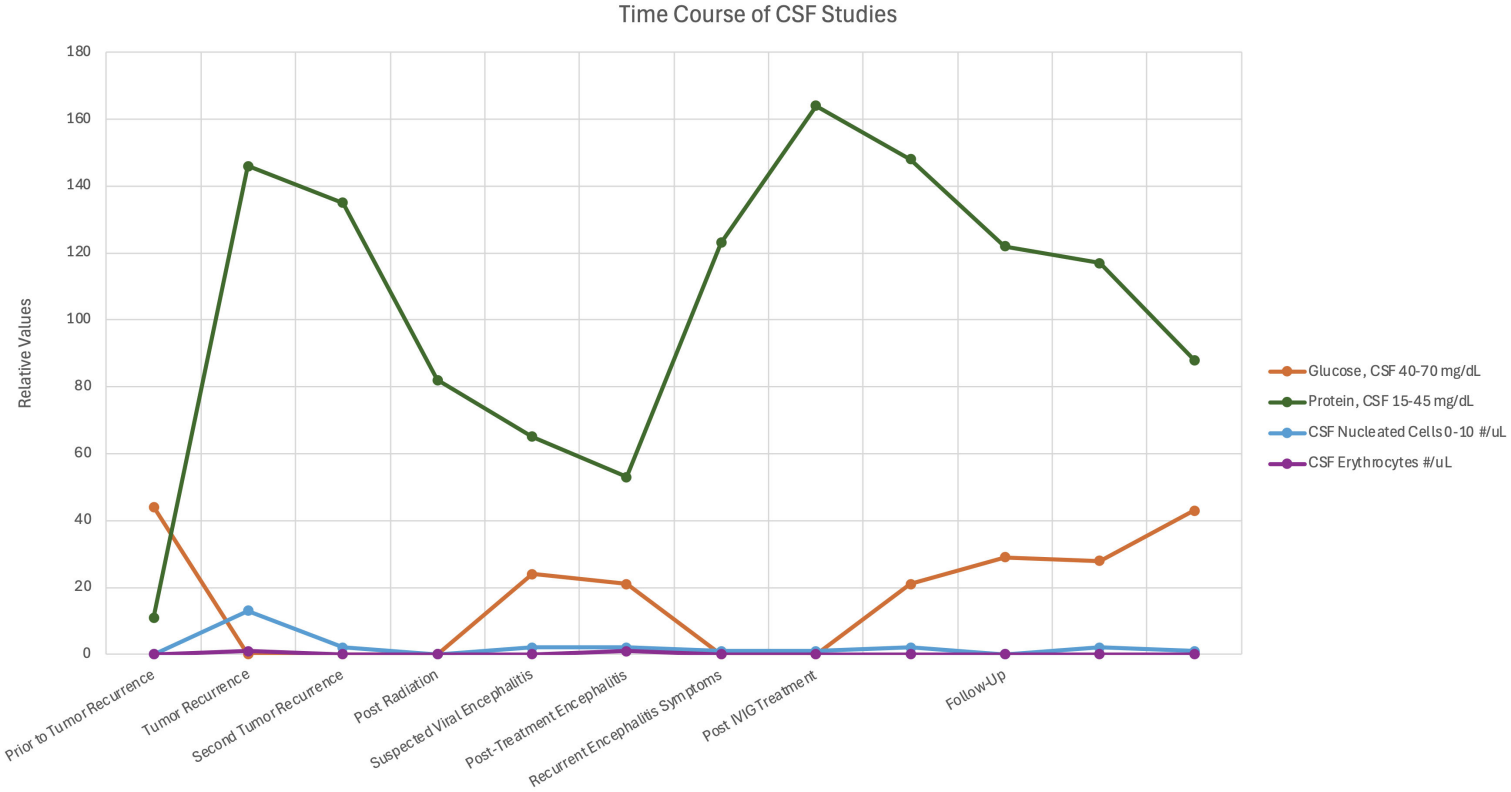
Time course of cerebrospinal fluid analysis associated during encephalitis. The graph depicts cerebrospinal fluid glucose, protein, and cell count during the encephalitis course.

The finding of ci-HHV-6 was later confirmed through nail clipping analysis, revealing paternal inheritance consistent with a diagnosis of ici-HHV-6B. Subsequently, the patient had several follow up cerebral spinal fluid testing which never demonstrated pleocytosis or positive cytology (time course shown in **Figure 2**) and repeat HHV-6B viral load testing from blood and CSF which remained elevated despite antiviral therapy consistent with an expected pattern in ici-HHV-6B cases where viral loads may reflect chromosomal integration rather than active replication.

**Figure 3:**
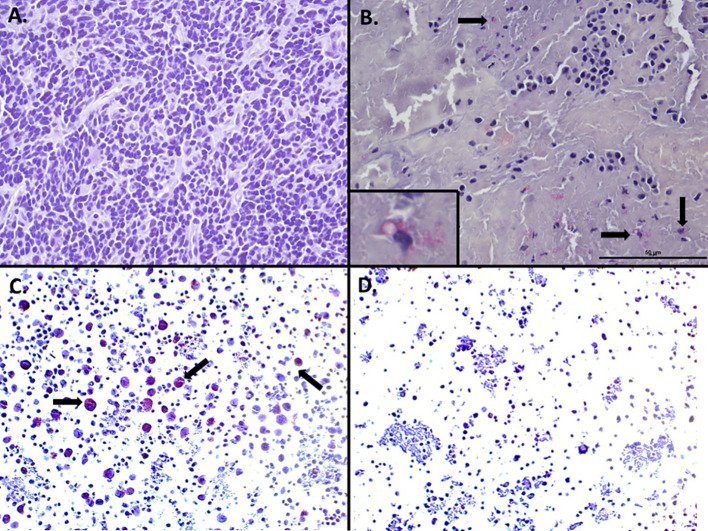
HHV-6 late glycoprotein H detection by immunohistochemistry in peritumoral lymphocytes in non-WNT non-SHH medulloblastoma. Immunohistochemistry of initial medulloblastoma did not reveal any staining for HHV-6 late glycoprotein H in the tumor cells **(A)**, however staining was identified in peritumoral lymphocytes in areas adjacent to the tumor (**B**, inset). HHV-6 infected (**C**, arrows) or uninfected **(D)** peripheral blood mononuclear cells served as positive and negative controls respectively.

Immunohistochemical analysis of the original medulloblastoma specimen revealed HHV-6 late structural glycoprotein (gH) antigen expression in peritumoral lymphocytes but not in the tumor cells (**Figure 3**). HHV-6B RT PCR serum analysis obtained one month post discharge demonstrated the presence of HHV-6 U100 glycoprotein Q mRNA (35.6 copies/10mL), supportive of active ici-HHV-6B though the temporal relationship to symptom onset remains unclear (**Table 1**). A short course of valganciclovir did not result in improvements in seizures or encephalopathy and was discontinued due to patient preference following two weeks. Twelve years post diagnosis of encephalitis, the patient remains tumor free without subsequent recurrent encephalitis. However, she never recovered from her pre-encephalitis baseline and has intellectual disability and intractable epilepsy.

## 3 Materials and methods

### 3.1 Immunohistochemical analysis

IHC was performed on formalin-fixed paraffin-embedded (FFPE) tissue samples using protocols as previously described by Coppe Laboratories. Briefly, FFPE slides were deparaffinized with xylene and rehydrated through decreasing gradients of ethanol. Antigen retrieval was performed using Proteinase K (20 ug/mL, 15 minutes, 37°C) enzymatic treatment. Slides were incubated with either a monoclonal antibody targeting a late structural glycoprotein (2 ug/mL HAR2 Mab reactive with HHV-6 envelope antigen gp86/110 of gH, BioWorld Consulting Laboratories, New Window, MD) of HHV-6 or an isotypic control antibody (2 ug/mL monoclonal antibody to KLH protein, Abcam, AB 18415-1001). Both primary and secondary antibodies were incubated at room temperature (20-24°C); primary Ab for 60 minutes and secondary Ab for 30 minutes. Detection was achieved using a biotinylated goat anti-mouse secondary antibody, Vectastain ABC-AP, and Vector Red AP reagents. Slides were counterstained with hematoxylin and bluing reagent before dehydration through increasing gradients of ethanol and cover slipping (23).

Control samples utilized human peripheral blood mononuclear cells mitogen stimulated with Phytohemagglutinin (PHA) and infected (positive control) or uninfected (negative control) with HHV-6B variant. Following development of cytopathic effects of approximately 30% in the HHV-6 infected culture both infected/non infected cells were fixed in 10% formalin followed by a standard paraffin embedding.

### 3.2 RNA reverse transcription quantitative PCR

RNA RT-qPCR was performed as previously described by the Department of Medicine, University of Washington (and Hill et al.). RNA was extracted from a PAXGene blood sample using MagMAX™ for Stabilized Blood Tubes RNA Isolation Kit (Ambion by Life Technologies). TURBO™ DNase treatment was included in the extraction process to minimize genomic DNA contamination. RT-qPCR was performed to detect the expression of HHV-6B genes U38, U90B, and U100B, with GAPDH as a control for RNA integrity assessment (GAPDH is a commercial assay designed across an exon-exon boundary and does not amplify genomic DNA).

Primers of U38, U90B, and U100B are listed below. Assays for U100 and U90 spanned splicing sites for RNA specificity, while U38 detects both RNA and DNA. No-reverse transcription (no-RT) PCR reactions were used as controls (24). The RT-PCR was amplified with Quantitec virus RT kit (QiaGen) using QuantStudio 7 Flex (Applied Biosystems by Thermal Fisher) under the thermal condition at 50°C for 20 minutes, then 95°C for 5 minutes, followed by 45 cycles of 60°C for 45 seconds and 95°C for 15 seconds. Detection of U38 in both no-RT and RT samples confirmed the assay’s ability to detect both RNA and DNA, validating that residual genomic DNA was present despite DNase treatment, which is consistent with the expected performance of this assay.

Primer and probe sequences used were as follows: ([forward],[reverse],[probe])

- U38: [TGCCCGATTYTGAAAAAGCT], [CCTGTGGGTATTCATAAAATTTTGC], [FAM_CTCCCGCGCTTTGCACAGACG-3’IBFQ]
- U90: [AGCAGGCTTTCAAAGGACACA], [CCCTCTGGAAACAACATGGAAT], [HEX-AACGTATGCAAAACTACCATC-MGB]
- U100: [GATAGTCTGTCCGCCATGGTTT], [CGCCAGAACTACAGACTGGAAA], [HEX-TCAGTTCGCATCGAGC-MGB]

### 3.3 Digital droplet PCR

Digital droplet PCR (ddPCR) was performed to determine HHV-6 type and estimate HHV-6 genome DNA copies per million cells, as previously described by Sedlak et al. The assay targeted a conserved 150 bp region of the U67 gene (present in both HHV-6A and HHV-6B) and included a non-conserved region for typing, using the human RPP30 gene as a cellular reference. Droplet digital PCR uses TaqMan chemistry similar to real-time PCR but partitions the reaction into thousands of individual droplets, allowing absolute quantification of DNA copies without the use of standard curves.

The ci-HHV-6 ddPCR was performed with ddPCR Supermix for Probes (Bio-Rad, Hercules, CA). Positive controls for HHV-6A and HHV-6B, negative cell controls, and non-template controls were included in each ddPCR run. DNA droplets were generated in a 96-well plate using the Bio-Rad Auto-Droplet Generator. PCR amplification was performed under thermal conditions of 94°C for 10 min, then 40 cycles at 94°C for 30 s and 60°C for 1 min, followed by 98°C for 10 min. Droplet reading was performed with the Bio-Rad QX200 Droplet Reader.

All wells required ≥10,000 accepted droplets to be considered valid. The positive cutoff was established at > 3 droplets. Results were analyzed using QuantaSoft Analysis Pro software. HHV-6 copies per cell were calculated using the formula: HHV-6 copies/(RPP30 copies/2), accounting for two copies of RPP30 in a diploid genome. Cross-reactivity between subtypes has been validated with no cross-reactivity (25, 26).

### 3.4 Chromosomal integration analysis

Chromosomal integration of HHV-6 was assessed using nail clipping samples, with methods previously described by Ward et al, with modifications as described by Coppe laboratories. Five or more nail clippings collected close to the nail plate were processed using a QIAamp DNA Investigator Kit #56504. Quantitative PCR was performed targeting the conserved HHV-6 U94gene and human RPP30 DNA as cellular genomic reference to distinguish between ci-HHV-6 and HHV-6 reactivation. Qualitative HHV-6A and HHV-6B PCR testing then determines the HHV-6 integrated species with results reported as “detected” or “not detected.” (18, 27, 28) Variability in cell content across nail samples represents a potential confounding factor in this analysis.

## 4 Discussion

### 4.1 Clinical significance

Our case report highlights a complex clinical scenario involving paternally inherited chromosomally integrated HHV-6B (ici-HHV-6B) in a pediatric medulloblastoma patient who developed encephalitis and subclinical seizures following proton therapy and prior history of high dose chemotherapy with autologous stem cell rescue. This is the first documented case of ici-HHV-6B in a pediatric patient with medulloblastoma and encephalitis where immunohistochemical (IHC) late structural antigen positivity was identified in peritumoral lymphocytes and RT PCR demonstrated U100 RNA transcripts, raising important questions about its potential role in CNS pathology.

Chromosomal integration of HHV-6 is unique as it is the sole human herpesvirus found to integrate into the genome, occurring in approximately 1% of the general population (2, 8, 18, 19). It is vertically transmitted in a Mendelian manner, as confirmed in our patient via nail clipping PCR analysis, which revealed paternal inheritance, and digital droplet PCR analysis, which confirmed marked elevation in viral loads consistent with ci-HHV-6. Studies have shown that ci-HHV-6 prevalence may be as high as 2% in certain populations – such as patients with encephalitis and pediatric transplant recipients – compared to 0.8% in the general population (18). This raises questions about whether ci-HHV-6B confers an increased risk or predisposition to CNS pathologies or other diseases, particularly in immunocompromised patients.

### 4.2 Viral biology

A well-established tropism for the central nervous system has been demonstrated by HHV-6, particularly the B subtype (3, 7, 15). In immunocompetent patients, primary HHV-6 infection, such as infantum roseola, can lead to febrile seizures and, in rare cases, encephalitis (1, 12–14). However, in immunocompromised patients, such as those undergoing hematopoietic stem cell transplantation (HSCT), HHV-6 reactivation can cause severe complications, particularly posttransplant acute limbic encephalitis (PALE) and post-procedural encephalitis in stem cell, bone marrow, and solid organ transplant recipients (4, 10, 11, 18). In cases of ci-HHV-6, reactivation has been reported in a child with X-linked severe combined immunodeficiency. In this case Endo et al. provided molecular and virologic evidence of ci-HHV-6A reactivation by RT-PCR late/immediate-early genes, immunostaining of immediate-early genes from bone marrow, and immunofluorescent staining of isolated HHV-6A from the patient’s peripheral blood mononuclear cells (29). Furthermore, they also sequenced the circulating strain and found that it matched the sequence of the integrated strain (29). While we did not use the same level of rigor in our approach to demonstrate activation of ici-HHV-6B, including viral sequencing to differentiate ici-HHV-6 from exogenously acquired HHV-6, several findings are suggestive of potential viral activity. The detection of U100 RNA transcripts from the blood and detection of late structural antigen of the patient’s medulloblastoma is supportive of activation. Unfortunately, we did not test the blood for HHV-6 RNA transcripts prior to the presentation of encephalitis and therefore do not know if HHV-6B RNA detection may have been pre-existing. The U100 RNA detection post-symptom onset may represent an epiphenomenon rather than a causative factor. However, the presence of late antigen in peritumoral lymphocytes from the original tumor specimen, obtained prior to immunosuppressive therapies and encephalitis presentation, suggests persistent viral protein expression associated with ici-HHV-6B.

### 4.3 Immunological context and treatment considerations

While the stem cell transplant for medulloblastoma therapy was autologous, making it a less likely cause for viral reactivation due to absence of graft-versus-host dynamics, the patient’s history of extensive immunosuppressive treatments may have contributed to viral reactivation and subsequent encephalitis. Radiation therapy induced reactivation of neurotropic herpesviruses has been reported and may be a plausible etiology of encephalitis (30). The presence of HHV-6 late antigen glycoprotein was present prior to chemotherapy, transplant, and radiation supportive of persistent ici-HHV-6B/e-HHV6B and worthy of further study. In order to definitively conclude that the HHV-6 U100 mRNA identified from our patient was from reactivation of ici-HHV-6 and not exogenous HHV-6, the transcript would need to be sequenced and compared to the ici-HHV-6 sequence. However, this testing is not available clinically at this time, highlighting the importance of recognition of ici-HHV and the use of clinical judgement when consideration of potential therapies.

The therapeutic response in our case requires careful interpretation. It is unclear whether the improvement in our patient’s clinical status was related to IVIG, ganciclovir or a combination of both. While clinical improvement appeared to follow IVIG and ganciclovir treatment, the persistent elevation of HHV-6B viral loads post treatment (1.12E+05 copies/cell) highlights the complexity of treating ici-HHV-6 cases. A previous report of a patient with persistent HHV-6 DNAemia and ci-HHV-6B with meningoencephalitis responsive to IVIG has been reported (32). The lack of decreased HHV-6B viral loads following treatment with valacyclovir and valganciclovir is not unsurprising as viral loads are irrelevant in cases of ci-HHV-6 as there is no way to differentiate active from latent viral DNA by qPCR. Unfortunately, the background copy number in ici-HHV6 is so high that even in patients with ici-HHV-6 there could be activated/reactivated infection that could be masked by the HHV-6 DNAemia. This suggests that any observed clinical benefit may reflect immunomodulatory effects rather than direct antiviral activity. Given the profound neurologic symptoms experienced by our patient, we decided empirical treatment with both IVIG and antiviral therapy was clinically appropriate despite the diagnostic uncertainties.

### 4.4 Tumor-virus relationship

The detection of HHV-6 late glycoprotein antigens in peritumoral lymphocytes and not tumor cells presents an intriguing pattern that differs from previous reports of HHV-6 in brain tumors, where viral detection has been reported in up to 60% of cases (2, 3, 15–17, 22). The concurrent detection of U100 glycoprotein Q mRNA indicates either active viral replication or the presence of eHHV-6. This finding demonstrates that ci-HHV-6B has the potential to reactivate under specific conditions and potentially contribute to clinical symptoms – a phenomenon that remains debated in literature (3, 18). Although HHV-6 has been implicated in CNS tumors, particularly low-grade gliomas, the absence of viral antigens within the tumor itself in our case limits direct tumorigenic implications (10). While previous studies suggest HHV-6 integration into telomeres may induce epigenetic modifications or alter subtelomeric gene expression (31), the specific relationship between ci-HHV-6 and brain tumor development remains hypothetical and requires further research (2, 6).

### 4.5 Diagnostic limitations and future directions

Several important limitations in our diagnostic approach must be acknowledged. One of the limitations of our diagnostic workup was the lack of testing for other autoimmune etiologies including Myelin oligodendrocyte glycoprotein antibody-associated disease (MOGAD), which has been associated with cerebral cortical encephalitis (33). Unfortunately, MOGAD testing was not clinically available at the time of diagnosis. An alternative diagnosis of MOGAD or other autoimmune encephalitis could explain the responsiveness to IVIG but does not detract from our findings. Even in cases of MOGAD, HHV-6 has been reported to co-occur with MOGAD in two reported cases that may suggest an association (34, 35). In the case of co-occurring MOGAD and HHV-6, Jumah et al. reported an HHV-6 plasma viral load of 14500copies/ml, which may have reflected ci-HHV-6 given the high copy number but was not tested. Another limitation of our workup was a lack of CSF HHV-6 IgM and IgG titers which may have distinguished an incidental ci-HHV-6 finding versus active infection superimposed on ci-HHV-6 since intrathecal antibody production may be a distinguishing feature between an incidental ci-HHV-6 finding and active infection superimposed on ci-HHV-6.

Despite extensive diagnostic evaluation, we cannot definitely establish causation between ci-HHV-6 and the patient’s symptomatology (6). High viral loads and the detection of viral RNA may represent either an incidental finding in the context of immunosuppression or indicate an active pathogenic process exacerbated by the patient’s recent radiation therapy. The lack of serial RNA and viral load testing post-IVIG and antiviral treatments prevents us from assessing the longitudinal effects of therapy and the absence of re-biopsy prevents conclusion of viral involvement in the patient’s tumor recurrence or progression.

### 4.6 Clinical implications and recommendations

Given the high seroprevalence of HHV-6 and the up to 2% prevalence of ci-HHV-6 in the general population, we suggest similar cases undergo comprehensive testing including whole blood PCR, digital droplet PCR or hair/nail PCR testing to confirm ici-HHV-6 status and variants. In cases where antiviral therapy is used, we suggest obtaining baseline and post-treatment HHV-6 viral titers and RNA detection (only available on a research basis) to determine the role of antiviral treatment, if any, in cases of ci-HHV-6 reactivation. Pre-transplant screening of immunocompromised patients and organ donors for ici-HHV-6 could improvement diagnostic accuracy by providing baseline integration status. Our case contributes to the diversity of literature of ici-HHV-6, highlighting the importance of ici-HHV-6 and the need to differentiate from active infection through copy number analysis. Future studies are needed to delineate the pathogenic versus incidental role of ci-HHV-6 in CNS diseases, and standardized diagnostic protocols could clarify the temporal relationship between ci-HHV-6 reactivation in CNS disease.

## Data Availability

The original contributions presented in the study are included in the article/supplementary material. Further inquiries can be directed to the corresponding author.
